# Ultrafast Response in AC-Driven Electrochemiluminescent Cell Using Electrochemically Active DNA/Ru(bpy)_3_^2+^ Hybrid Film with Mesoscopic Structures

**DOI:** 10.1038/s41598-017-09123-2

**Published:** 2017-08-17

**Authors:** Shota Tsuneyasu, Ryota Takahashi, Haruki Minami, Kazuki Nakamura, Norihisa Kobayashi

**Affiliations:** 1 Department of Image & Materials Science, Graduate School of Advanced Integration Science, Chiba University, 1-33 Yayoi-cho, Inage-ku, Chiba 263-8522 Japan; 2 Molecular Chirality Research Center, Chiba University, 1-33 Yayoi-cho, Inage-ku, Chiba 263-8522 Japan

## Abstract

Electrochemiluminescence (ECL) refers to light emission induced by an electrochemical redox reaction. The stability, emission response, and light intensity of the ECL device are known to be improved by using an alternating current (AC) voltage. In this paper, an AC-driven ECL device is fabricated with DNA/Ru(bpy)_3_^2+^ hybrid film-modified electrode. The Ru(bpy)_3_^2+^ complex exhibits significant electrochemical reactivity in the DNA/Ru(bpy)_3_^2+^ hybrid film prepared by electrochemical adsorption. The hybrid film contains unique micrometre-scale aggregates of Ru(bpy)_3_^2+^ in DNA matrix. The physicochemical properties of the hybrid film and its AC-driven ECL characteristics in the electrochemical device are studied. Orange-coloured ECL is observed to be emitted from only the aggregated structures in the hybrid film at the high AC frequency of 10 kHz, which corresponds to a response time shorter than 100 μs.

## Introduction

Electrochemiluminescence (ECL) is defined as the emission of light due to electrochemical redox reactions^1^. The best known mechanism for ECL generation is the annihilation pathway, which is based on the formation of excited molecular state due to electron transfer between the reduced and oxidised species^2–4^. The ECL system based on this pathway is expected to be a next-generation light emitting device^5–8^. In particular, such ECL devices could be fabricated into fibre-like^9^, curved/flexible^10, 11^, and arbitrarily shaped structures^12^. They can also be integrated with reflective display devices that utilise liquid crystal- or organic electrochromic-technologies to realise dual mode display^13–16^.

Among the reported ECL systems, the best studied material is the tris(2,2′-bipyridyl)ruthenium(II) ion (Ru(bpy)_3_^2+^). The corresponding ECL mechanism is known as follows (*designates electronically excited state)^17, 18^:

1 $${\rm{Ru}}{({\rm{bpy}})}_{3}{}^{2+}\to {\rm{Ru}}{({\rm{bpy}})}_{3}{}^{3+}+\,{{\rm{e}}}^{-}$$

2 $${\rm{Ru}}{({\rm{bpy}})}_{3}{}^{2+}+{{\rm{e}}}^{-}\to {\rm{Ru}}{{({\rm{bpy}})}_{3}}^{+}$$

3 $${\rm{R}}{\rm{u}}{({\rm{b}}{\rm{p}}{\rm{y}})}_{3}{}^{+}+{\rm{R}}{\rm{u}}{({\rm{b}}{\rm{p}}{\rm{y}})}_{3}{}^{3+}{\to }^{\ast }{\rm{R}}{\rm{u}}{({\rm{b}}{\rm{p}}{\rm{y}})}_{3}{}^{2+}+{\rm{R}}{\rm{u}}{({\rm{b}}{\rm{p}}{\rm{y}})}_{3}{}^{2+}$$

4 $${}^{\ast }{\rm{R}}{\rm{u}}{({\rm{b}}{\rm{p}}{\rm{y}})}_{3}{}^{2+}\to {\rm{R}}{\rm{u}}{({\rm{b}}{\rm{p}}{\rm{y}})}_{3}{}^{2+}+{\rm{h}}\upsilon $$

First, the reduced and oxidised species of Ru(bpy)_3_^2+^ are electrochemically generated at the cathode and anode, respectively. When these species collide with each other, electronic excitations occur. The ECL device is usually driven by the application of a direct current (DC) voltage. In this case, Ru(bpy)_3_^3+^ and Ru(bpy)_3_^+^ must diffuse into the bulk solution between the cathode and anode before colliding. Thus, the response time of ECL is not very short and depends on the thickness of the cell. In order to overcome this problem, we found that the ECL properties can be enhanced by using alternating current (AC)^19, 20^. Compared to the DC-driven system, the AC-driven counterpart does not require long-range diffusion of the reduced and oxidised species, because they are generated at the same electrode. Therefore, the deactivation of the generated active species during the long-range diffusion is suppressed, resulting in higher ECL intensities and decreased response time (several ms)^19–23^.

To further reduce the ECL response time for application in light emitting devices, the ECL materials could be immobilised at the molecular level onto the electrode surface, because this prevents the diffusion of the generated active species into the bulk. In this study, we used a functional DNA complex film to immobilise the redox active materials onto the electrode surface. Various functional materials, such as organic dyes, metal complexes, and conducting polymers are known to form complexes with DNA through electrostatic binding, intercalation, and groove binding^24–27^. Further, it was reported that the emission intensity is considerably improved by incorporating emissive molecules in the DNA matrix. Therefore, DNA-based functional materials have attracted great interest for applications in electronic and optical devices^28–31^. In this paper, we fabricated a novel DNA/Ru(bpy)_3_^2+^ hybrid film, and used it to modify the electrode to improve the ECL response. The resulting ECL device showed extremely high turn-on response (<100 μs).

**Figure 1:**
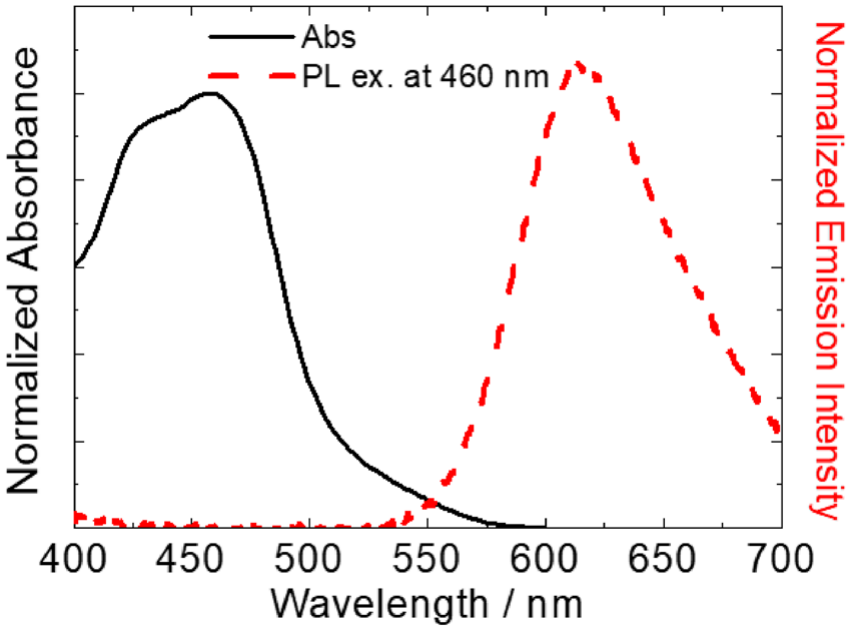
Absorption and photoluminescence spectra of DNA/Ru(bpy)_3_^2+^ hybrid film.

The absorption and photoluminescence spectra of the Ru(bpy)_3_^2+^-adsorbed DNA hybrid film were measured (Fig. 1). Metal-to-ligand charge transfer (MLCT) absorption and emission assignable to Ru(bpy)_3_^2+^ were clearly observed in the visible region, indicating that Ru(bpy)_3_^2+^ was incorporated in the DNA matrix. The absorption band of the incorporated Ru(bpy)_3_^2+^ was broadened compared to that in the propylene carbonate (PC) solution, although the spectral shape and peak wavelength hardly changed^32, 33^. Therefore, the DNA matrix did not affect the electronic structure of Ru(bpy)_3_^2+^. To estimate the amount of Ru(bpy)_3_^2+^ within the hybrid film, Ru(bpy)_3_^2+^ complex on the film was thoroughly dissolved in water, and the absorbance of Ru(bpy)_3_^2+^ at 455 nm (molar absorbance coefficient: ε = 10900 M^−1^ cm^−1^). The molar ratio of DNA (phosphate concentration): Ru(bpy)_3_^2+^ complex was estimated to be 6:1.

**Figure 2:**
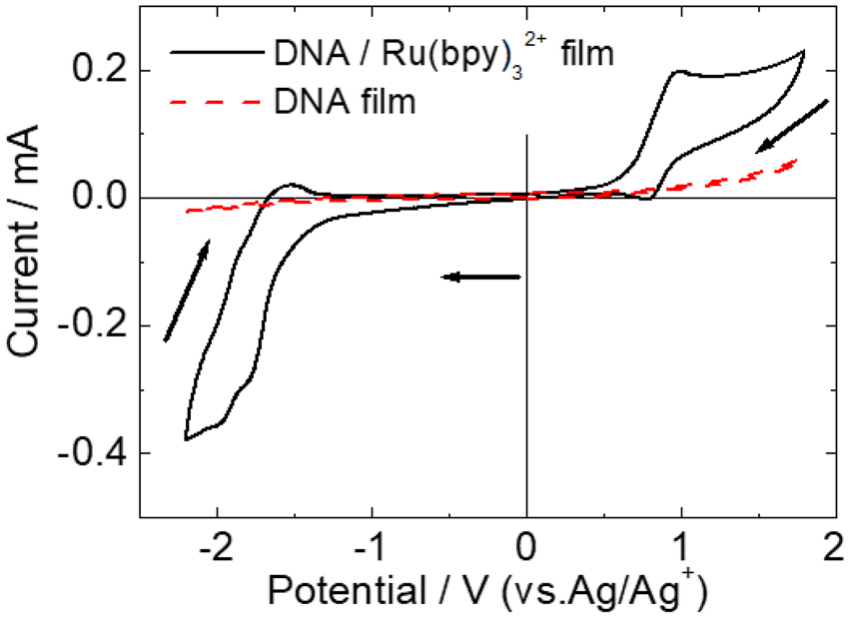
Cyclic voltammograms of DNA/Ru(bpy)_3_^2+^ and pure DNA films on an ITO electrode. Scan rate: 50 mV s^−1^.

Then, the electrochemical properties of the Ru(bpy)_3_^2+^-incorporated DNA film were investigated. Figure 2 shows the cyclic voltammetry (CV) curves of the DNA films with and without Ru(bpy)_3_^2+^ on an indium-tin oxide (ITO) electrode in PC solution containing tetra-n-butylammonium perchlorate (TBAP) electrolyte. For the pure DNA film, no significant electrochemical response was observed over the potential range between −2.2 V and +1.8 V (vs. Ag/Ag^+^). On the other hand, the first electrochemical oxidation peak of Ru(bpy)_3_^2+^ and the first reduction peak of Ru(bpy)_3_^2+^ were observed at around +1.0 V and −1.7 V, respectively. Such redox behaviour of Ru(bpy)_3_^2+^ complex in the hybrid film was almost the same as that in PC solution^32, 33^. Therefore, the incorporation into the DNA film did not affect the electrochemical activity and emission properties of Ru(bpy)_3_^2+^, suggesting that Ru(bpy)_3_^2+^ in the DNA film could generate ECL under a bias voltage.

**Figure 3:**
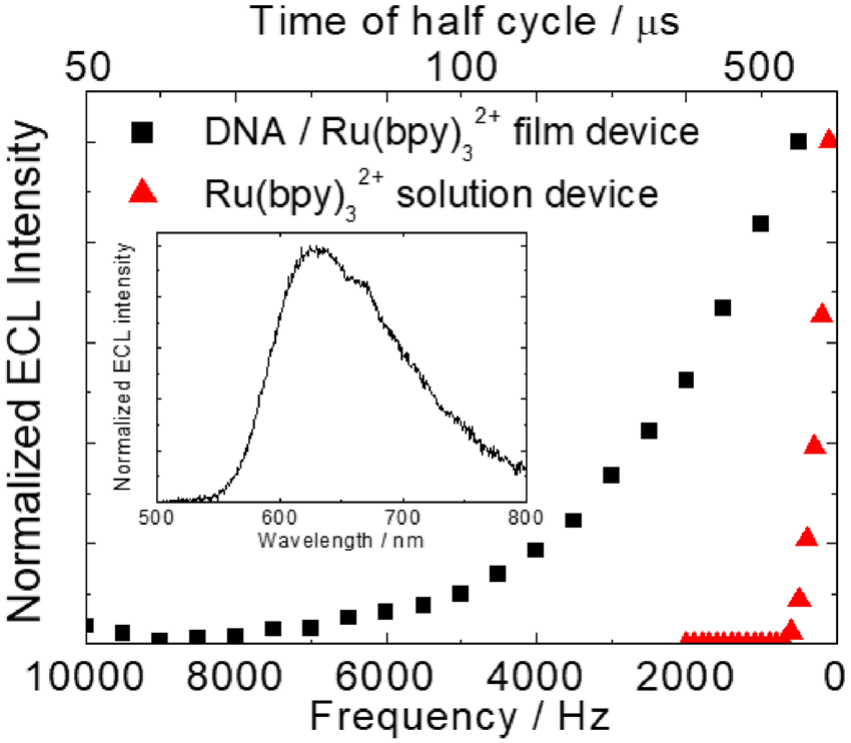
Frequency dependence of ECL intensity from the two devices under ±4 V AC voltage. Inset: ECL spectra of the devices under ±3 V, 50 Hz AC voltage.

An AC-driven ECL device was fabricated by sandwiching the electrolyte solution between a pair of hybrid film-modified ITO electrodes. For reference, another solution-based ECL device was fabricated by placing PC solution containing Ru(bpy)_3_^2+^ and TBAP between a pair of bare ITO electrodes. By applying an AC voltage of ±4.0 V at given frequencies, orange-colored ECL emission with a peak wavelength at 620 nm was observed in both devices (Fig. 3 inset). The generation of ECL from the Ru(bpy)_3_^2+^ complex indicates that the electrochemically oxidised and reduced species (Ru(bpy)_3_^3+^ and Ru(bpy)_3_^+^, respectively) collide with each other in the same film, leading to light emission. Figure 3 shows the frequency dependence of the normalised ECL intensities under ±4 V rectangular voltage. ECL from the solution-based device was only observed at frequencies below 500 Hz, which is similar to previous reports^20, 21^. On the other hand, in the DNA/Ru(bpy)_3_^2+^ hybrid film-based device, ECL was observed at frequencies as high as 10 kHz surprisingly. At higher frequency, the time in a half cycle was not enough to proceed the redox reaction of the ECL materials in comparison with that at lower frequency. From this reason, brightness of the ECL device at higher frequency was smaller than that at lower frequency. Therefore, the ECL intensity increased with decreasing AC frequency, with a luminance of approximately 1.0 cd/m^2^ at 500 Hz. Additionally, when the AC frequency decreased to 50 Hz, the luminance reached approximately 10 cd/m^2^. As just discribed, the AC-driven, DNA/Ru(bpy)_3_^2+^ hybrid film-based ECL device showed extremely fast turn-on response compared to the solution-based device, even though its maximum emission intensity was relatively lower. The maximum luminance in the hybrid film based ECL device was still smaller than that in solution based ECL device (~120 cd/m^2^ at 50 Hz^22^). One of the reasons is because effective electrode area for redox reaction on DNA/Ru(bpy)_3_^2+^-modified electrode was smaller than that on conventional flat ITO electrode. Due to the difference of mean electrode area, the amount of generated redox species on the DNA/Ru(bpy)_3_^2+^ film was thought to be quite smaller than that on conventional ITO electrode.

**Figure 4:**
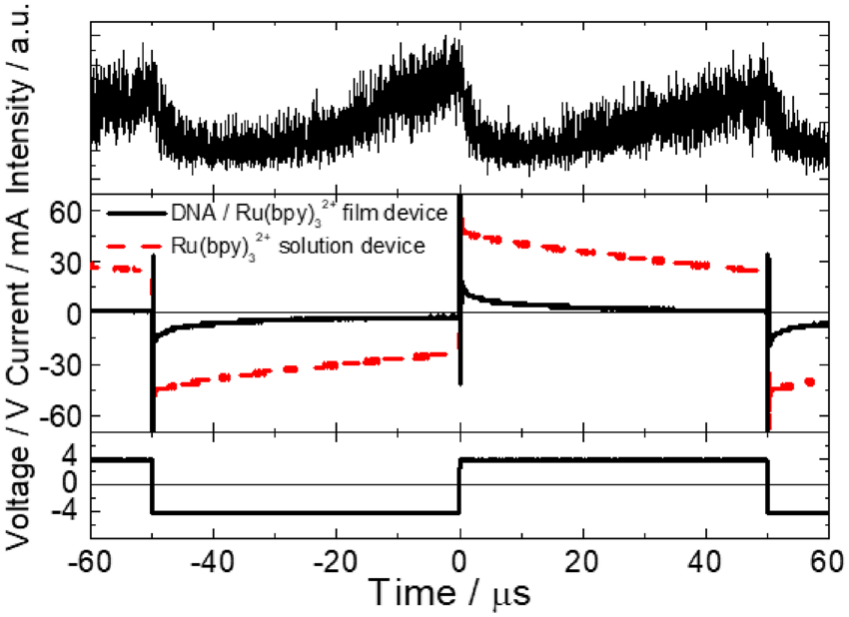
Transient ECL response (top) and transient current (middle) of the two devices under ±4 V, 10 kHz AC voltage (bottom).

Next, the transient ECL intensity and current response of the devices were investigated, in order to understand the origin of the quick ECL response (Fig. 4). Generally, when a bias voltage is applied to an electrochemical device, an electric double layer (EDL) is formed to induce the electrochemical redox reaction. In the solution-based conventional electrochemical device with an electrode area of 25 mm^2^, the experimentally measured time for charging the EDL is approximately 1 ms (since ECL was obtained only at frequencies below 500 Hz). The reason is that at 10 kHz, the corresponding half-cycle time (50 μs) is not sufficient for completely charging the EDL. In contrast, the EDL in DNA/Ru(bpy)_3_^2+^ hybrid film-based ECL device can be completely charged within 10 μs, based on the measured current response. Such quick charging of the EDL allows the subsequent redox reactions of Ru(bpy)_3_^2+^ in the film during the next AC cycle. The continuous rectangular AC wave caused Ru(bpy)_3_^3+^ and Ru(bpy)_3_^+^ to collide with each other to form the excited states of Ru(bpy)_3_^2+^, thereby generating ECL. The quick charging of the EDL film is thought to be key to the fast ECL response (less than 100 μs) in the DNA/Ru(bpy)_3_^2+^ hybrid film device.

**Figure 5:**
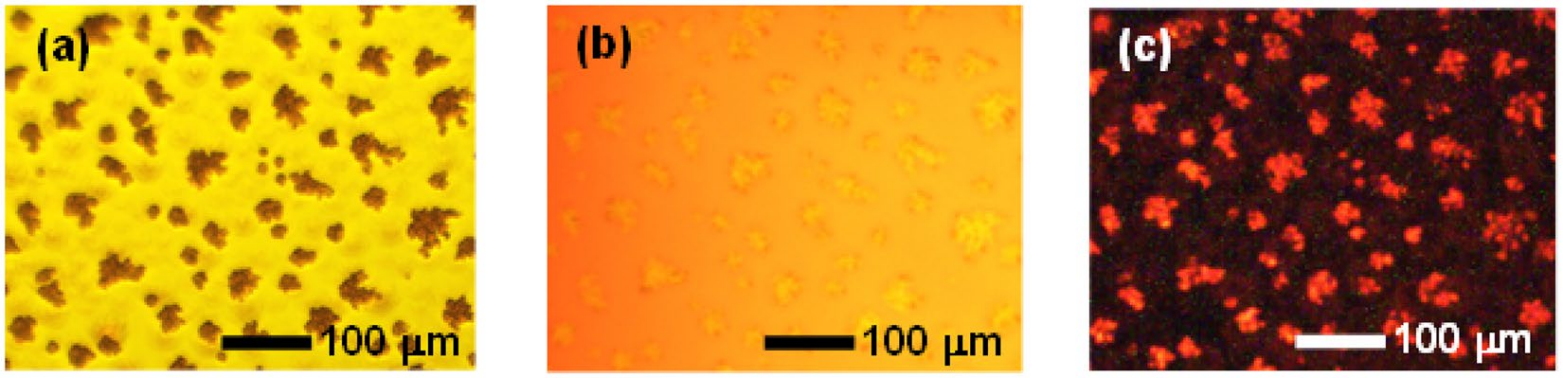
(**a**) Microscopic and (**b**) fluorescence microscopic images of DNA/Ru(bpy)_3_^2+^ hybrid film, showing the flat and aggregated regions. (**c**) Microscopic images of the film under ±4 V, 500 Hz AC voltage in the ECL device. Photoexcited fluorescence spectra of the ECL device upon excitation by a 455 nm laser.

We further utilised an optical microscope to view the membrane structure of the hybrid film. As shown in Fig. 5(a)–(c) the film contains flat parts and embedded micro-aggregated parts. The fluorescence micrograph of the hybrid film (Fig. 5(b)) indicates that Ru(bpy)_3_^2+^ was distributed all over the film. However, ECL emission under AC voltage was only observed in the aggregated parts (Fig. 5(c)). Therefore, the flat and aggregated parts have different electrochemical responses. From the optical microscopic observation of the DNA/Ru(bpy)_3_^2+^ film, such kind of micro-aggregation array was automatically formed during the drying process of the DNA film. Mechanism on formation of specific array of the aggregations with mesoscopic scale is not clear at present. However, such structure was not found in other polyanion films such as Nafion, Flemion, and polystyrene sulfonate. In these films, amount of the aggregations of Ru(bpy)_3_^2+^ were quite small and non-uniformly distributed in comparison with DNA/Ru(bpy)_3_^2+^ film. Further, the size of aggregations in these films were smaller than that in DNA/Ru(bpy)_3_^2+^ film. During incorporation process of Ru(bpy)_3_^2+^, much amount of Ru(bpy)_3_^2+^ could penetrate into the DNA film, which is swollen in water solution. The penetrated Ru(bpy)_3_^2+^ was thought to be effectively stored in DNA film because the Ru(bpy)_3_^2+^ could be binded in DNA though several binding modes such as electrostatic interaction to phosphate groups, intercalation, groove binding and so on. Those interactions between Ru(bpy)_3_^2+^ and DNA enable to incorporate large amount of the Ru(bpy)_3_^2+^ uniformly. Much amount of the Ru(bpy)_3_^2+^ electrophoretically incorporated in the DNA film would lead to formation of specific array of Ru(bpy)_3_^2+^ aggregates under drying process.

**Figure 6:**
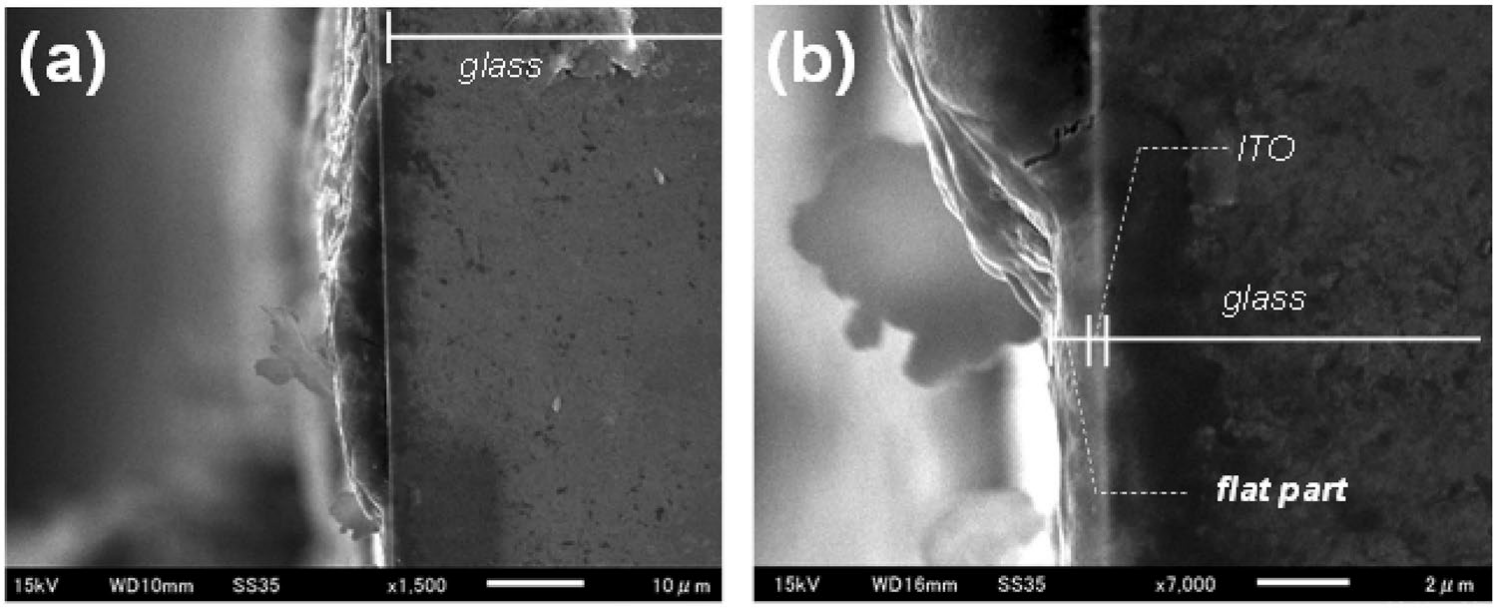
Cross-sectional SEM images of DNA/Ru(bpy)_3_^2+^ hybrid film at (**a**) low (**b**) high magnifications.

**Figure 7:**
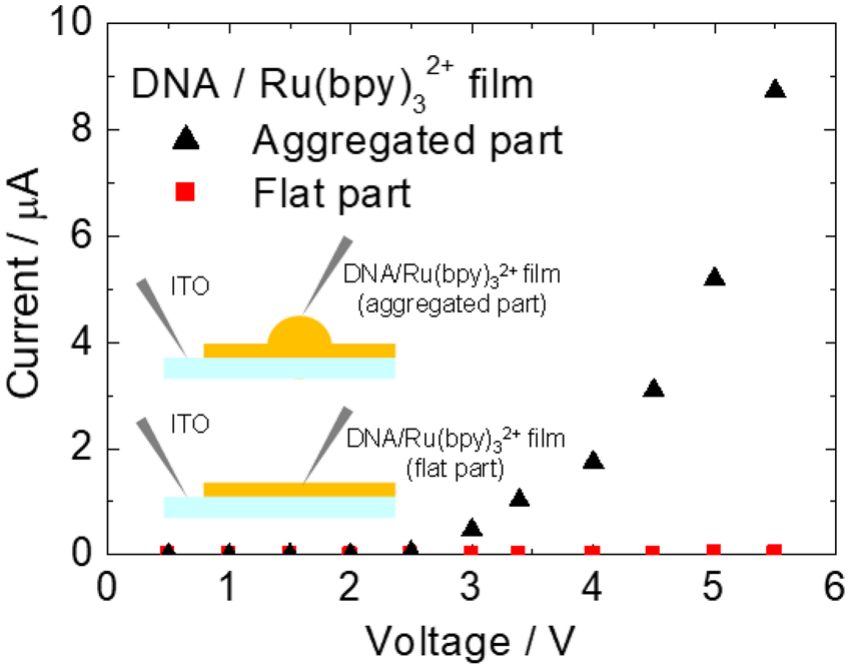
I–V characteristics of the flat and aggregated parts of DNA/Ru(bpy)_3_^2+^ hybrid film on ITO. Inset: experimental setup with probe needles.

The cross-sectional scanning electron microscopy (SEM) image of DNA/Ru(bpy)_3_^2+^ hybrid film at low magnification (Fig. 6(a)) also shows the existence of flat and aggregated parts, and the thickness of the latter was approximately 5 μm. Under higher magnification (Fig. 6(b)), the thickness of the flat region is approximately 1 μm. To understand the different electrochemical responses of the two parts, we measured the I-V characteristics by using a probe needle (Fig. 7). A higher current was observed for the aggregated part, even though this part was about five times thicker than the flat part. According to previous reports^34, 35^, fast turn-on ECL response was observed on artificially fabricated single microelectrode. The turn-on response time of an electrochemical cell with a 5 μm radius microelectrode was approximately 3 μs^35^. This quick electrochemical response can be explained in terms of effect of double layer capacitance and ohmic drop. As the results of the effects, the redox reaction of the ECL materials on the microelectrode was induced even at high frequency. In this research, we utilized the DNA/Ru(bpy)_3_^2+^ hybrid film to the AC-driven ECL device. The aggregated structures containing Ru(bpy)_3_^2+^ which was approximately 5 to 20 μm radius were distributed all over the DNA film (Fig. 5), and the density of the aggregates was approximately 100~150 pieces per mm^2^. In addition to this, the electrochemical response in the aggregated parts were higher than that in the flat part (Fig. 7). From transient current response, the electrical double layer in the ECL device with Ru(bpy)_3_^2+^ hybrid film was completely charged within 10 μs. Moreover, the ECL from Ru(bpy)_3_^2+^ was observed only in the aggregated part. Thus, the aggregated parts in the film were thought to work as microelectrode array that lead to the quick charging of electrical double layer, leading to the achievement of the ultrafast response in ECL.

**Figure 8:**
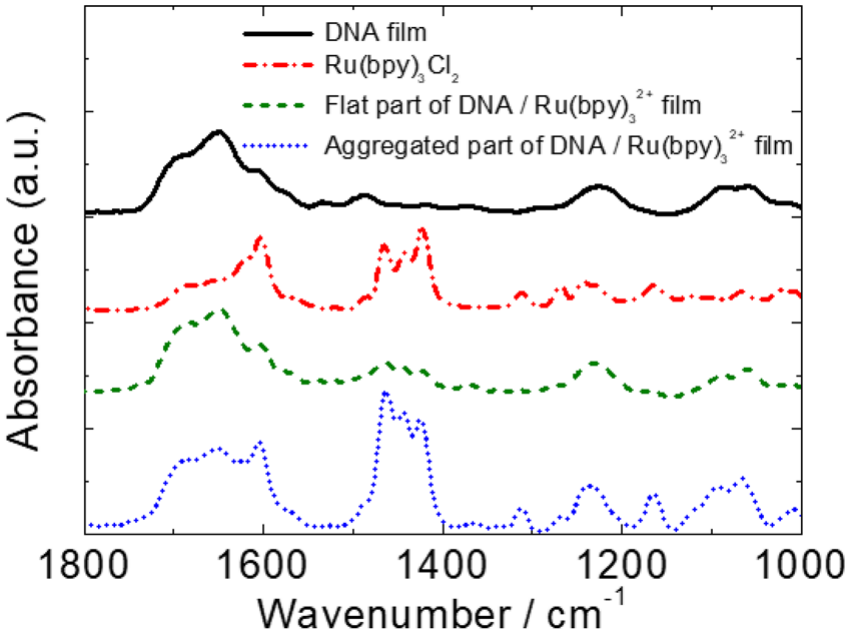
Micro FT-IR spectra of the DNA/Ru(bpy)_3_^2+^ hybrid and pure DNA films on ITO electrode, and the spectrum of Ru(bpy)_3_Cl_2_.

Finally, we measured the microscopic Fourier transform infrared spectroscopy (FT-IR) spectra of Ru(bpy)_3_Cl_2_, pure DNA, and the flat and aggregated parts of the hybrid film to analyse the membrane structure in detail. In Fig. 8, the peaks at 1085 and 1224 cm^−1^ for both the flat and aggregated parts of DNA/Ru(bpy)_3_^2+^ hybrid film are assignable to PO_2_^−^ asymmetric and symmetric stretching vibrations. These peaks were blue shifted, indicating Ru(bpy)_3_^2+^ electrostatically interacts with the anionic phosphate group. The absorption intensities at 1420–1463 and 1605 cm^−1^, which are assignable to Ru(bpy)_3_^2+^, were higher for the aggregated part than for the flat part. Further, absorption peaks assignable to phosphate groups were also observed in the aggregated part. These spectral features indicate that the aggregated regions in the film consist of both Ru(bpy)_3_^2+^ and DNA, and with a higher Ru(bpy)_3_^2+^ content than in the flat part. These DNA-Ru(bpy)_3_^2+^ microaggregates are essential for the fast ECL response.

We fabricated DNA/Ru(bpy)_3_^2+^ hybrid films with a mesoscopic aggregated structure. The corresponding AC-driven ECL device showed extremely fast turn-on response in comparison to the solution-based device. The ECL emission from the DNA/Ru(bpy)_3_^2+^ hybrid film only comes from the aggregated parts of the film, which possibly act as microelectrodes and allow the quick charging of the EDL. In order to obtain ECL with such a quick response, the generated oxidised and reduced states of Ru(bpy)_3_^2+^ have to diffuse from the electrode because oxidised (reduced) species generated in the first half-cycle should be easily reduced (oxidised) in the following half-cycle, if the diffusion is not so fast. We are currently working on clarifying the reason behind the excellent electrochemical responses of these DNA/Ru(bpy)_3_^2+^ aggregates, and the function of DNA in them.

## Experimental

Ru(bpy)_3_Cl_2_ (Tokyo Chemical Industry Co. Ltd.), tetra-*n*-butylammonium perchlorate (TBAP; Kanto Chemical Co. Inc.), and PC (Kanto) were chosen as the electrochemiluminescent material, supporting electrolyte, and solvent, respectively. They were used as received. 10k bps DNA (phosphate group concentration) was provided by Ogata Research Lab Co. Ltd.

First, a native DNA film (thickness: 0.5 μm) was prepared by casting the DNA solution on an ITO electrode. Then, Ru(bpy)_3_^2+^ was introduced by placing the film in a Ru(bpy)_3_Cl_2_ aqueous solution (10 mmol/L), and then applying −1.5 V (*vs*. Ag/Ag^+^) voltage to the DNA film electrode. In this step, there was no supporting electrolyte in the solution. Therefore, Ru(bpy)_3_^2+^ was likely moved by migration and was incorporated into the DNA film through electrostatic interaction. The ECL device was prepared by placing a PC solution containing TBAP (100 mM) between a pair of DNA/Ru(bpy)_3_^2+^ hybrid film-modified electrodes placed 75 μm apart.

Ultraviolet-visible (UV-vis) absorption spectra of the samples were measured using a spectrophotometer (JASCO Co., V-570). Photoluminescence spectra were obtained using a spectrofluorometer (JASCO, FP-6600). CV was performed using an electrochemical analyser. A three-electrode cell was constructed with the ITO-based working electrode, Pt wire as the counter electrode, and an Ag/Ag^+^ electrode as reference.

The AC voltage was applied to the ECL devices by a function generator (Iwatsu Electric Co., Ltd., SG-4115). ECL spectra were measured using a photonic multichannel analyser (Hamamatsu Photonics, PMA C10027). The ECL emission responses were detected with a photomultiplier tube (Hamamatsu, H10721-20MOD) and signal amplifier (Hamamatsu, C9663), and monitored with an oscilloscope (Teledyne LeCroy, Wavejet 314). Fluorescent microscopic observations were made under a polarising microscope (Olympus, BX51-P). The surface morphology of the DNA/Ru(bpy)_3_^2+^ film was characterised through SEM (JEOL Ltd., JSM-6510). FT-IR analysis was carried out with a spectrophotometer (JASCO, FT/IR-410).
